# Effects of Iodonium Analogs on Nadph Oxidase 1 in Human Colon Cancer Cells

**DOI:** 10.3390/antiox10111757

**Published:** 2021-11-03

**Authors:** Krishnendu K. Roy, Jiamo Lu, James H. Doroshow

**Affiliations:** 1 Division of Cancer Treatment and Diagnosis, National Cancer Institute, National Institutes of Health, Bethesda, MD 20892, USA; royk@nci.nih.gov; 2 Center for Cancer Research, National Cancer Institute, National Institutes of Health, Bethesda, MD 20892, USA; lujiamo@mail.nih.gov

**Keywords:** NADPH oxidase 1 (NOX1), diphenyleneiodonium (DPI), di-2-thienyliodonium (DTI), reactive oxygen species (ROS), superoxide (O_2_^●^^−^), hydrogen peroxide (H_2_O_2_)

## Abstract

Recent studies suggest that of the molecules postulated to function as inhibitors of the NADPH oxidase family of enzymes iodonium analogs known to broadly interfere with flavin dehydrogenase function demonstrate mechanistic validity as NADPH oxidase poisons. In recent work, we have produced a series of novel iodonium compounds as putative inhibitors of these oxidases. To evaluate the potential utility of two novel molecules with favorable chemical properties, NSC 740104 and NSC 751140, we compared effects of these compounds to the two standard inhibitors of this class, diphenyleneiodonium and di-2-thienyliodonium, with respect to antiproliferative, cell cycle, and gene expression effects in human colon cancer cells that require the function of NADPH oxidase 1. Both new agents blocked NADPH oxidase-related reactive oxygen production, inhibited tumor cell proliferation, produced a G1/S block in cell cycle progression, and inhibited NADPH oxidase 1 expression at the mRNA and protein levels at low nM concentrations in a fashion similar to or better than the parent molecules. These studies suggest that NSC 740104 and NSC 751140 should be developed further as mechanistic tools to better understand the role of NADPH oxidase inhibition as an approach to the development of novel therapeutic agents for colon cancer.

## 1. Introduction

The seven members of the NADPH oxidase (NOX) family, NOX1-5 and dual oxidases (DUOX) 1 and 2, are essential transmembrane enzymes that are widely expressed across normal and malignant tissues [1]. By generating either O_2_^●^^−^ (NOX1, 2, 3, and 5) or H_2_O_2_ (NOX4 and DUOX1 and 2), these proteins play a critical role in the maintenance of host defense, the initiation of an inflammatory response, cytokine signaling, angiogenesis, vascular tone, and cellular proliferation [2,3,4,5,6]. There is accumulating evidence that pre-malignant conditions (including inflammatory bowel disease and pancreatitis) [7,8] that occur as a consequence of chronic, unrestrained inflammation may, at least in part, be mediated by NOX family oxidase-dependent production of reactive oxygen species (ROS) [9].

Among normal human tissues, NOX1 is most abundantly expressed by epithelial cells found in both the small and large intestine [10]. Generation of superoxide by NOX1 requires the presence of two regulatory subunits, NOXO1 (NOX organizer 1) and NOXA1 (NOX activator 1) that are homologs of p47*^phox^* and p67*^phox^*, respectively; these proteins are critical for the activation of the NOX1 catalytic subunit and assembly with p22*^phox^* to promote transmembrane electron flow. Using our recently developed specific NOX1 mouse monoclonal antibody that recognizes the C-terminal region of the NOX1 protein, we confirmed that approximately one-third of human colon cancers and one-half of pre-malignant colonic adenomas express NOX1 protein at a high level; in both conditions, NOX1 expression is significantly greater than that observed in the normal colon [11]. Not only is NOX1 frequently overexpressed in human colon cancers, but recent studies have demonstrated that an inflammatory milieu, characterized by the presence of pro-inflammatory cytokines in the colorectal cancer microenvironment [12], may play an important role in the progression of these malignancies. Thus, it is highly relevant that we have recently demonstrated NOX1 expression in human colon cancer cells to be dramatically up-regulated by a variety of the same pro-inflammatory molecules present in the colon cancer matrix [13].

To evaluate the relevance of NOX1 expression for the growth of colonic cancers, we examined the effect of stable genetic knockdown of NOX1 expression on human colon cancer cells both in vitro and in vivo [4]. In these experiments, we found that HT-29 cells in which NOX1 expression was inhibited by shRNA demonstrated a profound decrease in ROS production as well as a significant decrease in proliferation both when cultured in vitro and when propagated as xenografts carried by athymic mice. NOX1 inhibition was accompanied by a block in cell cycle progression at the G1/S interface, diminished cell signaling along the MAPK pathway, and a significant increase in protein-tyrosine phosphatase activity.

Based on these results, NOX1 appears to be an authentic molecular target that is a suitable focus for the development of small molecule therapeutic agents to treat colon cancer [14]. Thus, it is not surprising that substantial prior effort has been directed toward developing such agents [15]; unfortunately, it has recently been demonstrated that essentially all of the molecules advanced to date as putative NOX inhibitors lack molecular specificity for any NOX isoform [16]. In the latter effort, we found that only molecules of the iodonium class could be demonstrated to specifically interfere with the enzymatic production of ROS by NOX species. Unfortunately, the two ‘parent’ molecules of the iodonium class, diphenyleneiodonium (DPI) and di-2-thienyliodonium (DTI), although capable of authentic NOX inhibition, are also well known to interfere with the function of a wide range of flavin dehydrogenases; these two compounds also suffer from poor solubility and other suboptimal physical properties. For these reasons, we recently developed a series of novel iodonium molecules that might have improved properties as NOX inhibitors [11]. In the experiments reported here, we have compared the properties of two novel iodonium class molecules, NSC 740104 and NSC 751140, to the parental compounds, DPI and DTI, to evaluate their potential utility as NOX1 inhibitors. We found that not only are these novel compounds highly potent inhibitors of the function of NOX1, but they also inhibit the expression of NOX1 mRNA and protein in human colon cancer cells and block human growth hormone-induced phosphorylation of the c-Met oncoprotein and interleukin-4 mediated upregulation of NOX1 as well as Stat6 signaling.

## 2. Materials and Methods 

### 2.1. Reagents and Drugs

**Figure 1:**
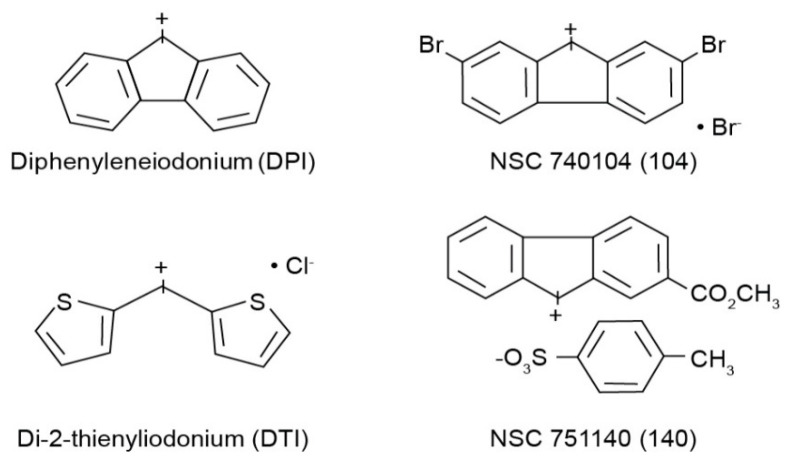
Structures of the four iodonium analogs examined in our experiments.

All cell culture medium and reagents were from Grand Island Biological Co. (Grand Island, NY, USA). DPI was purchased from Sigma-Aldrich (St. Louis, MO, USA), while DTI was obtained from Color Your Enzyme (Bath, ON, Canada). Other DPI analogs used in this work were synthesized by the Developmental Therapeutics Program, Division of Cancer Treatment and Diagnosis of the National Cancer Institute (Bethesda, MD, USA) as previously described [14]; the structures of these molecules are shown in Figure 1. DPI and its analogs were dissolved before use in dimethyl sulfoxide. Unless otherwise noted, all other chemicals were from Calbiochem (San Diego, CA, USA). Antibodies against total and phosphorylated proteins were purchased from Cell Signaling Technologies (Beverly, MA, USA); CS# 3129 for phosphorylated Met; CS# 3077 for total Met; CS# 56,554 for phosphorylated-Stat6; CS# 5397 for Stat6. Antibody against human β-actin (cat# A3853) was from Sigma-Aldrich. HGF was from R & D Systems Cat# 294-HGN. Goat anti-rabbit IgG-HRP (sc-2004), and goat anti-mouse IgG-HRP (sc-2005) were from Santa Cruz Biotechnology (Dallas, TX, USA). Antibodies were used for Western analysis as described previously [13]. The mouse anti-human monoclonal antibody against human NADPH oxidase 1 (NOX1) was developed and characterized by our laboratory as recently described [11]. For these experiments we employed precast gels from Invitrogen by Thermo Fisher Scientific: Novex^™^ wedge well 4–20% Tris-Glycine gels (1.0 mm × 10 and × 15 well), catalog #XP04200BOX. We obtained nitrocellulose membranes from Invitrogen by Thermo Fisher: iBlot 2NC Regular Stacks, catalog #IB23001.

### 2.2. Cell Culture

HT-29, SW-620, and KM-12 human colon cancer cell lines were obtained from American Type Culture Collection (Manassas, VA, USA) and cultured in RPMI medium supplemented with 10% fetal bovine serum (Grand Island Biological Co.). All cell cultures were maintained at 37 °C in a humidified atmosphere containing 5% CO_2_. Cells were split and passed into new flasks at a time of sub-confluence; the cells were used for studies while in logarithmic phase growth. 

### 2.3. Expression of NOX1, NOXA1, and NOXO1 mRNA

Cells were cultured in 100-mm Petri dishes with an initial cell number such that 60–80% confluence would be achieved after 48 h. RNA was isolated and quantitative analysis of NOX1, NOXO1 and NOXA1 mRNA levels were performed using real-time RT-PCR as previously detailed [4,11].

### 2.4. Reactive Oxygen Species Production

Cells were cultured in 60-mm Petri dishes and allowed to grow to 60–80% confluence. The cells were exposed to the molecules of interest for 4 h; exogenous H_2_O_2_, where examined, was applied for 5 min. ROS production in the presence of DPI and its analogs was compared to untreated control samples. The cells were harvested by trypsinization and washed twice with 1x PBS buffer. Cells were resuspended in 1x PBS and incubated at 37 °C with 10 mM 2′-7′dichlorodihydroflourescein diacetate acetyl ester, H2DCF-DA (Molecular Probes Inc., Eugene, OR), in the dark for 30 min. The samples were then analyzed by flow cytometry, FACSCalibur (Becton Dickinson Immunocytometry Systems, San Jose, CA, USA), utilizing CellQuest software (Becton Dickinson Immunocytometry Systems) as previously described [14]. For some studies, NOX1-related ROS production was also measured using a luminol-based superoxide anion kit from Sigma-Aldrich exactly as described in a recent publication [11]. 

### 2.5. Growth Inhibition Assay

Cells were cultured in 100-mm Petri dishes with an initial cell number such that 60–80% confluence would be achieved after 48 h. These exponentially growing cells were exposed to various concentrations of DPI, DTI, or DPI analogs for 48 h. The media was collected and stored on ice; adherent cells were harvested with trypsin. The previously removed medium was then used to neutralize the trypsin, and the cells were collected by centrifugation at 2000 rpm for 5 min. Trypan Blue (0.4%) staining was used to enumerate viable cells using a hemocytometer for the growth inhibition experiments with DPI and DTI. The effects of a 48-h exposure to the iodonium analogs NSC 740140 and NSC 740104 on the proliferation of HT-29 cells were measured using an MTT cell growth assay as described [14]. 

### 2.6. Cell Cycle Analysis

Tumor cells were cultured in 60-mm Petri dishes and allowed to grow to 50-60% confluence. The cells were then exposed to the molecule of interest for 24 h and 48 h and compared to control samples not exposed to the investigational compounds. Following exposure, the cells were harvested with trypsin and centrifuged at 2000 rpm. The harvested cells were washed twice with 1x PBS and then resuspended and fixed in 70% ethanol/1x PBS added in pulses while vortexing. Cell samples were stored at 4 °C for a minimum of 6 h and a maximum of 2 months prior to analysis. When samples were to be analyzed, the ethanol was removed by centrifugation, and the cell pellets were resuspended in 50 µL of propidium iodide stain (10 mg/mL propidium iodide, 100 mg/mL RNAse in PBS). The samples were incubated for 30 min at room temperature in the dark. Cell cycle distribution was determined by analytical cytometry utilizing a FACS caliber flow cytometer (BD Biosciences, San Jose, CA, USA). Data from experiments performed in triplicate were analyzed with ModFit v3.0 software. 

### 2.7. BrdU Incorporation

Cells were cultured in 60-mm Petri dishes and allowed to grow to 50–60% confluence. The cells were then exposed to the molecule of interest for 24 h and compared to control unexposed samples. Prior to harvesting with trypsin, the cells were incubated at 37 °C with 20 µM BrdU (BrdU kit; Sparta Labs, Biocarta San Diego, CA, USA) for 1 h in the dark. The cells were harvested, washed twice, and resuspended with 1x PBS. Cells were activated with UVB light for 5 min and then fixed with 70% cold ethanol for 24 h prior to evaluation. For analysis, the samples were first made permeable by washing in 0.5% Tween-20/PBS solution, and subsequently washed with 1x PBS. The samples were then resuspended in deionized water and fluorescence-activated cell sorting buffer (provided with the kit). To each sample, anti-BrdU FITC and 7-amino-actinomycin D DNA staining reagent were added followed by brief vortexing. The cells were incubated at room temperature for 1 h in the dark; stained cells were subsequently analyzed using a FACS Calibur Flow Cytometer (Becton Dickinson, San Jose, CA, USA) equipped with a 488-nm laser capable of detecting both 7-AAD and FITC. 

### 2.8. Western Analysis

Cells were cultured in 120-mm Petri dishes and allowed to grow to 60–80% confluence. The cells were then washed twice with 1x PBS, and lysed for 10 min at 4 °C in a buffer containing 50 mM Tris-HCl (pH 7.4), 1% Nonident P-40, 2 mM EDTA, 2 mM, EGTA, 150 mM NaCl, 100 mM NaF, 1 mM sodium vanadate, 0.5% sodium deoxycholic acid, and a Complete Mini protease inhibitor cocktail tablet (Roche Diagnostics, Mannheim, Germany). The lysates were cleared by centrifugation at 13,000 rpm for 30 min at 4 °C. Total protein concentrations were measured using bicinchoninic acid reagent (Pierce, Rockford, IL, USA) and prepared with 0.5% beta-mercaptoethanol in SDS Protein Gel Solution (Pierce). For Western blotting, protein samples were separated through precast SDS-polyacrylamide gels and transferred onto nitrocellulose membranes at 4 °C overnight. Membranes were blocked with 5% nonfat, dry, instant milk in TTBS (150 mM NaCl, 10 mM Tris, pH 7.8, 0.01% Tween-20) for 1 h and incubated with the primary antibody for 2 h in TTBS. The membranes were then treated with horseradish peroxidase-conjugated secondary antibody for 1 h in 5% milk and developed using an ECL kit (Amersham Biosciences, Piscataway, NJ, USA).

### 2.9. Statistical Analyses

Data have been expressed as the mean ± standard deviation of 3 independent experiments. Statistical differences were assessed with Student’s *t* test; NS, not significant, *p* > 0.05.

## 3. Results

### 3.1. Basal Expression of NOX1, NOXO1, and NOXA1

Because the goal of these studies was to examine the effects of a series of iodonium analogs on NOX1, we evaluated the baseline expression of each component of the NOX1 complex in three different human colon cancer cell lines. As demonstrated in Table 1, we found that the HT-29 cell line expressed all three NOX1 components, whereas both KM-12 and SW620 cells lacked detectable amounts of two of the three subunits of the NOX1 complex, and thus would not be expected to generate NOX1-related ROS.

### 3.2. Effect of Iodonium Analogs on ROS Production by Human Colon Cancer Cells

**Figure 2:**
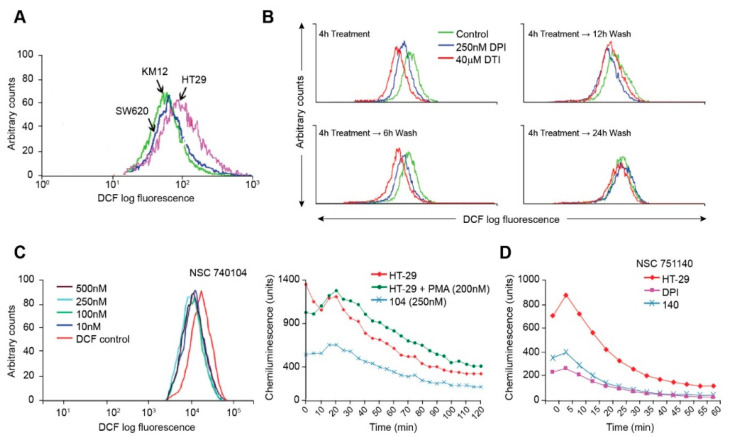
Inhibition of ROS production by iodonium analogs. Baseline ROS production for HT-29, KM-12, and SW620 human colon cancer cell lines is shown in (**A**) and the effects of DPI and DTI on ROS formation and recovery from treatment following removal of the drugs is demonstrated in (**B**). The effects of NSC 740104 on ROS formation in HT-29 cells are shown in (**C**). The left panel in section (**C**) demonstrates the concentration-dependent effects of NSC 740104 on ROS formation in HT-29 cells evaluated by DCF fluorescence. In the right panel the effect PMA on ROS production from NOX1 in HT-29 cells is shown as well as the inhibition of baseline ROS production in HT-29 cells by NSC740104 using an orthogonal luminescence technique to measure superoxide. The effects of NSC751140 and DPI on superoxide production determined by luminescence in HT-29 cells is demonstrated in (**D**). These results are representative of at least three experiments.

Using the redox-sensitive fluorescent probe H_2_DCF-DA, we assayed the basal levels of ROS produced by the colorectal carcinoma cell lines. In the presence of ROS, H_2_DCF-DA is cleaved, and the resultant molecules fluoresce upon laser activation. We found, as expected, that the HT-29 cell line produced substantively more baseline DCF fluorescence than either KM-12 or SW620 cells (Figure 2A), suggesting that NOX1-related ROS increase the steady-state level of reactive oxygen formation in HT-29 cells beyond that expected from other sources, including the mitochondrial electron transport chain.

Using the fluorescent probe H_2_DCF-DA, we also determined that DPI and DTI decreased ROS production in a reversible manner. In HT-29 cells, intracellular ROS levels were markedly decreased in the presence of DPI and DTI after a 4 h treatment (Figure 2B). Inhibition of ROS production was reversible; native ROS levels were gradually regained following removal of DPI or DTI (following the 4 h treatment), washing of the cells, and incubation with fresh complete culture media for increments of between 6 and 24 h (Figure 2B). DPI or DTI exposures, at 250 nM or 40 µM, respectively, produced negligible loss of cell viability, as determined by Trypan Blue staining. As a positive control, ROS levels were shown to increase with the addition of H_2_O_2_ into cell media in a concentration-related fashion (data not shown).

We also examined the effects of two novel iodonium analogs on ROS production by HT-29 cells. NSC740104 produced dramatic inhibition of DCF fluorescence at a concentration as low as 10 nM (Figure 2C); Figure 2C also reveals that ROS production by NOX1 in HT-29 cells is activated by phorbol myristate acetate, as previously described [11], and that NOX1 inhibition by NSC740104 is also demonstrable using a chemiluminescent assay for ROS. Figure 2D shows that pretreatment of HT-29 cells with a fourth iodonium analog, NSC 751140, at a concentration of 500 nM, also dramatically decreased ROS formation.

### 3.3. Effect of Iodonium Analogs on Human Colon Cancer Cell Growth

**Figure 3:**
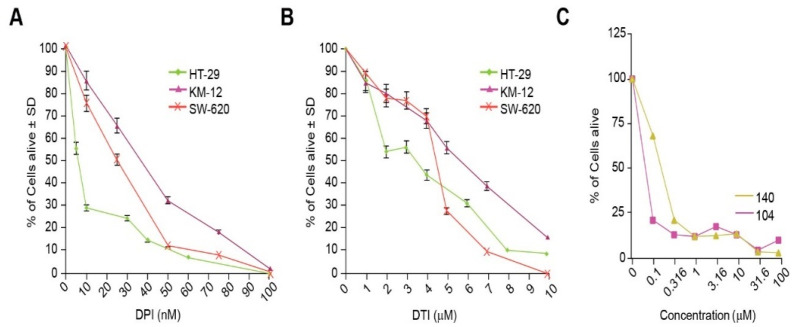
Inhibition of tumor cell growth by iodonium analogs. The effect of DPI and DTI on the proliferation of HT-29, KM-12, and SW620 colon cancer cells is shown in (**A**,**B**). Panel (**C**) demonstrates the concentration-dependent effects of NSC 751140 and NSC 740104 on the growth of HT-29 human colon cancer cells; mean data representative of triplicate experiments are shown. The effects of the two DPI analogs were not assessed in KM-12 and SW-620 cells because of their lack of NOX1 expression. In some cases, the error bars are too small to be observed (**C**).

The effect of the small molecules DPI and DTI as single agents on the growth of the colorectal carcinoma cell lines HT-29, KM-12, and SW-620 was assessed after 48 h of analog exposure. Treatment of HT-29 cells resulted in IC_50_ (concentration which produces a 50% reduction in cell growth) values of 10 nM for DPI and 3 µM for DTI. The IC_50_ values for KM-12 cells were 40 nM for DPI and 6 µM for DTI, while the IC_50_ values for SW-620 cells were 25 nM and 5 µM for DPI and DTI, respectively (Figure 3A,B). NSC 751140 and NSC 740104 also potently diminished HT-29 tumor cell growth at sub-micromolar concentrations (Figure 3C). 

### 3.4. Alterations in Cell Cycle Progression Produced by Iodonium Analogs

**Figure 4:**
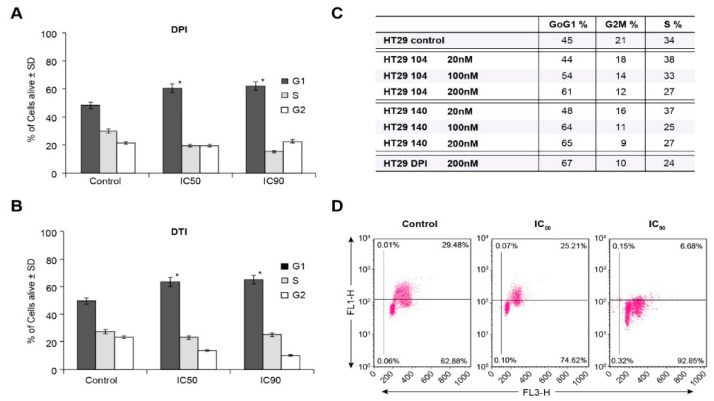
HT-29 G1/S cell cycle block produced by iodonium analogs. DPI and DTI inhibit cell cycle progression into S phase at their IC_50_ and IC_90_ concentrations (**A**,**B**). NSC 751140 and NSC 740104 also produce a G1/S cell cycle block in HT-29 cells at nM concentrations (**C**). Measurement of S-phase progression using BrdU incorporation confirms the decrease in S-phase cells produced by DPI in HT-29 cells. The FL1 channel represents BrdU incorporation, and the FL3 channel used 7-amino-actinomycin D as a measure of DNA, both expressed in arbitrary units (**D**). *, *p* < 0.005. Results are representative of three experiments.

To examine the basis for inhibition of cell growth resulting from iodonium analog exposure, we evaluated the effect of these compounds on cell cycle progression in HT-29 cells. Concordant with our growth inhibition data, DPI and DTI exposure at the ≈IC_50_ concentrations of 10 nM and 4 µM, respectively, resulted in a block at the G1/S cell cycle interface at the expense of the G2/M phase following a 24 h iodonium exposure (Figure 4A,B). Cell cycle inhibition on a 24 h time course was also observed for the IC_90_ concentrations of these compounds (60 nM and 10 µM for DPI and DTI, respectively). Parallel results were observed in a concentration-dependent manner for a 24 h exposure of HT-29 cells to the iodonium analogs NSC 751140 and NSC 740104 (Figure 4C). 

To confirm the ability of DPI to inhibit S-phase progression, measurement of BrdU incorporation was performed. Figure 4D demonstrates an inverse correlation between increasing DPI concentration (IC_50_ and IC_90_) and resulting decrements in S-phase incorporation for 24 h iodonium analog treatments.

### 3.5. Effects of Iodonium Analogs on NOX1 Expression, c-MET Phosphorylation, and IL-4-Related NOX1 Expression

**Figure 5:**
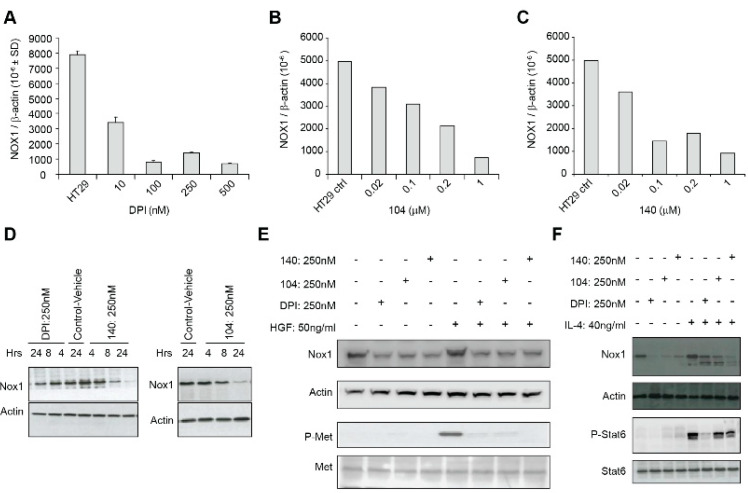
NOX1 expression and c-Met phosphorylation are decreased by io-donium analogs. Concentration-dependent inhibition of NOX1 mRNA ex-pression in HT-29 cells is shown for a 24 h exposure to DPI (**A**), a 24 h exposure to NSC 740104 (**B**), and a 6 h treatment with NSC 751140 (**C**). Decreased NOX1 expression at the protein level is demonstrated following treatment of HT-29 cells with DPI (250 nM), NSC 751140 (250 nM), or NSC 740104 (250 nM) for the time periods shown (**D**). Treatment of HT-29 cells with 250 nm DPI, 104 or 140 for 24 h blocked HGF-induced c-Met phosphorylation; HGF treatment was for 24 h, concurrent with iodonium analog exposure (**E**). Exposure of HT-29 cells to DPI, 104, or 140 (250 nM) for 24 h and to IL-4 (40 ng/mL) beginning 30 min after the iodonium analogs for a total of ~24 h decreased IL-4-mediated enhance-ment of NOX1 protein expression (**F**). These data are representative of three experiments.

In a previous study, we found that exposure of LS174T human colon cancer cells to DPI (250 nM) or DTI (10 µM) for 24 h produced a significant (>80%) inhibition of NOX1 mRNA expression without altering the expression levels of the antioxidant enzymes catalase or glutathione peroxidase [17]. To expand these findings, we evaluated the effect of iodonium analogs on NOX1 mRNA and protein expression in HT-29 cells. We found that DPI and NSC 740104 treatment both led to a concentration-dependent reduction in NOX1 mRNA levels without producing a concomitant decrease in cell viability (Figure 5A,B). Next, we evaluated the effect of NSC 751140 on NOX1 mRNA expression after a 6 h exposure; in the absence of altered cell killing, this iodonium analog also decreased NOX1 mRNA levels at low nM concentrations (Figure 5C). Using our newly described NOX1 monoclonal antibody, we confirmed that DPI, NSC 740104, and NSC 751140 treatment of HT-29 cells all decreased NOX1 expression at the protein level, even at early time points following iodonium exposure (Figure 5D).

It has been previously demonstrated that c-Met, when activated by hepatocyte growth factor (HGF) binding and tyrosine auto-phosphorylation, leads to cellular proliferation, propagation, and scattering. Over-activation of c-Met is a known hallmark of oncogenesis. To understand potential mechanisms by which iodonium analogs could exert their antineoplastic effects, and because studies have demonstrated an effect of ROS on HGF/c-Met signaling and cellular migration [18,19], we assessed the expression and phosphorylation state of c-Met following HGF exposure in the presence of DPI, 104, and 140 (Figure 5E). Exposure to a 250 nM concentration of DPI or its analogs for 24 h resulted in substantive loss of phosphorylation of c-Met concomitant with a decrease in NOX1 protein expression. We also found that iodonium analog exposure also decreased the IL-4-mediated increase in NOX1 expression that we had previously demonstrated [13] (Figure 5F). 

## 4. Discussion

The goal of the experiments reported herein was to compare the effectiveness of two novel iodonium analogs, NSC 751140 and NSC 740104 (Figure 1), to DPI and DTI as inhibitors of NOX1. Our previous experiments suggested that NSC 751140 and NSC 740104 were as potent or more potent, respectively, than DPI as inhibitors of the ability of NOX1 to generate O_2_^●^^−^ in a NOX1 overexpression model cell line system [11]. Because both of these two investigational molecules are predicted based on their chemical structures to possess enhanced physical properties compared to either DPI or DTI, we chose to directly evaluate the effects of the new compounds on colon cancer cell growth, cell cycle progression, and NOX1 expression versus established iodonium analogs known to specifically inhibit NOX1 [16].

We first evaluated the ability of three colon cancer cell lines to generate ROS based on their expression of the components of the NOX1 complex (Table 1 and Figure 2). As expected, HT-29 human colon cancer cells produce more ROS at baseline than either SW620 or KM-12 cells, since only this cell line possesses a full complement of the NOX1 subunits that are required for oxidase activity (Table 1). We also found that following a 4 h treatment both DPI and DTI inhibited intracellular ROS formation (Figure 2B). However, within 6–12 h of removing either iodonium analog, the inhibitory effects of the agents began to reverse; ROS inhibition was eliminated within 24 h of removal of either compound. As previously proposed [20], and as we previously demonstrated [11], iodonium analogs form adducts with the FAD portion of NOXs, probably mediated by the generation of phenyl radicals produced by electron transfer to the iodonium ring from the reduced NOX-associated flavin. Our findings suggest that the half-life of this adduct could be ≈ 6-12 h. Of the two novel iodonium analogs examined, NSC 740104 appears to be the more potent inhibitor of NOX1 in HT-29 cells, demonstrating low nM inhibitory activity (Figure 2C), as was previously demonstrated for a model cell line genetically reconstituted to express functional NOX1 [11].

Stable genetic inhibition of NOX1 with shRNA significantly diminishes the proliferation of HT-29 cells that possess functional NOX1, but not of the HCT116 human colon cancer line that lacks an active NADPH oxidase [4]. Thus, it is not surprising that the IC_50′_s for both DPI and DTI were substantively lower in NOX1-proficient HT-29 cells versus the SW620 or KM-12 NOX1-deficient lines (Figure 3A,B). Furthermore, we found that iodonium analogs produced a significant G1/S block in cell cycle progression at low nM concentrations, confirmed for DPI by concentration-dependent inhibition of BrdU incorporation (Figure 4A–D). Thus, the antiproliferative effects we found for these compounds, which occur over the same concentration range as that required to inhibit traverse into S phase, may be due, at least in part, to the G1 block we observed. These results are consistent with the G1 blockade, and concomitant loss of tumor cell proliferation, that we found previously for HT-29 cells wherein NOX1 function was inhibited with shRNA.

We also examined whether iodonium analogs, in addition to producing FAD adducts that interfere with electron transfer to oxygen across tumor cell membranes, might have other direct effects on NOX1 expression. As shown in Figure 5, DPI, NSC 751140, and NSC 740104 all produce a concentration-dependent inhibition of NOX1 mRNA expression in HT-29 cells. This occurs at the same low nM concentrations of these compounds observed for growth inhibition and altered cell cycle progression. Inhibition of NOX1 protein expression was also observed for DPI, NSC 740104, and NSC 751140 (Figure 5D). It is of interest that decreases in NOX1 mRNA expression and NOX1 protein expression were found as early as 4–8 h following iodonium exposure. While regulation of NOX1 expression by its interactions with its cognate subunits NOXA1 and NOXO1, by transcription factors and numerous cytokines, as well as by phosphorylation [21], and other post-translational modifications (such as by Hsp90 and SUMO1) have been described [1,22,23], inhibition of the process of NOX1 transcription itself by multiple iodonium analogs has not been demonstrated previously. It could be hypothesized that the rapid time course of this effect suggests a potential effect on NOX1 mRNA stability. Our laboratory has begun to examine this effect, which could provide important insights into the mechanism of action of the iodonium compounds on NOX species; preliminary evidence indicates that inhibition of NOX1 mRNA expression by iodonium analogs can be prevented by pre-incubation of HT-29 cells with actinomycin D, supporting a transcriptional event underlying this process (data not shown).

In light of our previous demonstration of the effects of NOX1 knockdown on MAPK signaling [4] that is due, in part, to enhanced activity of protein tyrosine phosphatases occurring secondary to decreased levels of intracellular ROS, we evaluated other ROS-dependent signaling pathways that play a critical role in tumor cell proliferation. As shown in Figure 5E, exposure to iodonium analogs markedly decreases c-Met phosphorylation initiated by HGF. Altered c-Met signaling by iodonium analogs in colon cancer cells has not been demonstrated previously. Inhibition of c-Met phosphorylation occurs concomitant with decreased NOX1 expression. c-Met dephosphorylation following iodonium exposure, as is the case for dephosphorylation of components of the MAPK cascade, could be due to the enhanced protein tyrosine phosphatase activity observed in HT-29 cells following NOX1 knockdown as described above. Furthermore, substantive effects of ROS on c-Met transactivation, signaling, and cell motility have been described previously [18,19]. In any case, c-Met dephosphorylation produced by iodonium analogs could provide an additional mechanism underlying the antiproliferative action produced by this class of compounds.

Finally, we demonstrated in Figure 5F that the Stat6-dependent increase in NOX1 expression that we have previously reported following IL-4 exposure in HT-29 cells [13] is partially blocked by iodonium analogs. Since NOX1 upregulation by IL-4 produces a proangiogenic milieu for HT-29 cells, this previously undescribed effect of the iodonium analogs could also be involved in their antiproliferative effects in vivo [17].

## 5. Conclusions

In conclusion, these experiments have demonstrated that several iodonium class molecules can inhibit NOX1 activity and expression in human colon cancer cell lines. Loss of NOX1-dependent ROS production is associated with diminished tumor cell proliferative capacity [24,25] that may be due, in part, to a block at the G1/S cell cycle interface. In addition, iodonium analogs have been shown for the first time in HT-29 cells to diminish NOX1 mRNA and protein levels, to decrease HGF-mediated c-Met phosphorylation, and to inhibit IL-4-related enhancement of NOX1 expression partially. These studies expand the mechanistic range of the inhibitory effects on NOX1 produced by iodonium analogs. 

## Figures and Tables

**Table 1:** Expression of the components of the NOX1 complex in human colon cancer cells.

NOX1 Component	HT-29	SW620	KM-12
NOX1 ^1^	4800	<50	<150
NOXO1	3500	<50	<50
NOXA1	1000	1000	7000

^1^ mRNA expression of NOX1, NOXO1, and NOXA1 normalized to β-actin × 10^−6^; mean values for three experiments that varied by <20%; please see [4,11,13] for detailed methods.
